# Muscle injury-induced hypoxia alters the proliferation and differentiation potentials of muscle resident stromal cells

**DOI:** 10.1186/s13395-019-0202-5

**Published:** 2019-06-19

**Authors:** Geneviève Drouin, Vanessa Couture, Marc-Antoine Lauzon, Frédéric Balg, Nathalie Faucheux, Guillaume Grenier

**Affiliations:** 1 Centre de Recherche du CHUS, 12e Avenue Nord, Sherbrooke, QC J1H 5N4 Canada; 2 Agriculture and Agri-Food Canada, 2000 College Street, Sherbrooke, QC J1M 0C8 Canada; 3 Laboratory of 3D Cell Culture Systems, Department of Chemical and Biotechnological Engineering, Faculty of Engineering, Université de Sherbrooke, 2500 Boul Universite, Sherbrooke, QC J1K 2R1 Canada; 4 Laboratory of Cell-Biomaterial Biohybrid Systems, Department of Chemical and Biotechnological Engineering, Faculty of Engineering, Université de Sherbrooke, 2500 Boul Universite, Sherbrooke, QC J1K 2R1 Canada; 5 Department of Orthopedic Surgery, Faculty of Medicine, Université de Sherbrooke, 12e Avenue Nord, Sherbrooke, QC J1H 5N4 Canada

**Keywords:** Heterotopic ossification, Damaged muscle, Hypoxia, BMP, Multipotent differentiation

## Abstract

**Background:**

Trauma-induced heterotopic ossification (HO) is a complication that develops under three conditions: the presence of an osteogenic progenitor cell, an inducing factor, and a permissive environment. We previously showed that a mouse multipotent Sca1^+^ CD31^−^ Lin^−^ muscle resident stromal cell (mrSC) population is involved in the development of HO in the presence of inducing factors, members of the bone morphogenetic protein family. Interestingly, BMP9 unlike BMP2 causes HO only if the muscle is damaged by injection of cardiotoxin. Because acute trauma often results in blood vessel breakdown, we hypothesized that a hypoxic state in damaged muscles may foster mrSCs activation and proliferation and trigger differentiation toward an osteogenic lineage, thus promoting the development of HO.

**Methods:**

Three- to - six-month-old male C57Bl/6 mice were used to induce muscle damage by injection of cardiotoxin intramuscularly into the tibialis anterior and gastrocnemius muscles. mrSCs were isolated from damaged (hypoxic state) and contralateral healthy muscles and counted, and their osteoblastic differentiation with or without BMP2 and BMP9 was determined by alkaline phosphatase activity measurement. The proliferation and differentiation of mrSCs isolated from healthy muscles was also studied in normoxic incubator and hypoxic conditions. The effect of hypoxia on BMP synthesis and Smad pathway activation was determined by qPCR and/or Western blot analyses. Differences between normally distributed groups were compared using a Student’s paired *t* test or an unpaired *t* test.

**Results:**

The hypoxic state of a severely damaged muscle increased the proliferation and osteogenic differentiation of mrSCs. mrSCs isolated from damaged muscles also displayed greater sensitivity to osteogenic signals, especially BMP9, than did mrSCs from a healthy muscle. In hypoxic conditions, mrSCs isolated from a control muscle were more proliferative and were more prone to osteogenic differentiation. Interestingly, Smad1/5/8 activation was detected in hypoxic conditions and was still present after 5 days, while Smad1/5/8 phosphorylation could not be detected after 3 h of normoxic incubator condition. BMP9 mRNA transcripts and protein levels were higher in mrSCs cultured in hypoxic conditions. Our results suggest that low-oxygen levels in damaged muscle influence mrSC behavior by facilitating their differentiation into osteoblasts. This effect may be mediated partly through the activation of the Smad pathway and the expression of osteoinductive growth factors such as BMP9 by mrSCs.

**Conclusion:**

Hypoxia should be considered a key factor in the microenvironment of damaged muscle that triggers HO.

**Electronic supplementary material:**

The online version of this article (10.1186/s13395-019-0202-5) contains supplementary material, which is available to authorized users.

## Background

Heterotopic ossification (HO) is defined as the formation of bone in soft tissue [1]. Clinical manifestations include increased joint stiffness, limited range of motion, swelling, and pain, all of which can result in severe functional limitations [2]. Although there is an uncommon hereditary disease called fibrodysplasia ossificans progressiva (FOP) that causes HO, most cases result from brain or spinal cord injury (“neurogenic HO”, NHO), local trauma such as orthopedic surgery, muscular trauma, fractures, severe burns (“traumatic HO”), or both processes [3–7]. Chalmers et al. proposed that three conditions must be met for the development of HO: the presence of a permissive environment, an osteogenic progenitor cell, and an inducing factor [8].

An increasing body of evidence points to hypoxia as a key element of the permissive environment [9–12] as it is involved in the induction of endochondral bone formation [13, 14]. Hypoxia activates the endochondral osteogenesis pathway via hypoxia-inducible factors (HIF) such as the HIF-1α and HIF-1β subunits. HIF-1α levels are dependent on oxygen concentrations. In normoxia, HIF-1α is degraded by the proteasome, while, in hypoxia, it accumulates in the cytoplasm and then translocates to the nucleus to form a complex with constitutively expressed HIF-1β. HIF-1α/HIF-1β complexes then bind to the hypoxia-responsive element (HRE) in the promoters of target genes such as VEGF and upregulate their transcription [15]. The upregulation of VEGF activates local angiogenesis, which serves as an early stepping stone for endochondral ossification [16–18]. HIF-1α may also be involved in HO and FOP [9–11]. For example, a recent study confirmed that FOP lesions occur in tissues in hypoxic conditions [11]. In addition, Lin et al. used a model of tenotomy-induced HO in Sprague-Dawley rats to show that the inhibition of HIF-1α expression prevents the formation of HO [9].

Several candidates such as Tie2^+^ cells, Tie2-Cre-lineage-labeled multipotent cells in the muscle interstitium (CD45^−^ CD31^−^ PDGFRα^+^ Sca1^+^), have been suggested to play a role in the development of traumatic HO [19–21]. We have also shown that Sca1^+^ CD31^−^ Lin^−^ muscle resident stromal cells (mrSCs) are able to differentiate in vitro into osteoblasts and to contribute in vivo to trauma-induced HO in the presence of inducing factors such as exogenous BMP2 or BMP9 [19]. In addition, we found that BMP9 causes HO only in cardiotoxin (CTX)-damaged skeletal muscle, suggesting a permissive microenvironment that modulates the response to BMP9 is required to induce mrSCs to form HO. We have also recently shown that BMP9 is present in human HO and is endogenously synthesized by osteoblasts and stromal cells [22].

Various experimental models of muscle damage have been used to show that there is a significant decrease in pO_2_ in damaged areas in the first hours and days following injury [23–25]. We thus hypothesized that hypoxia might affect the phenotype of mrSCs by promoting their activation and proliferation and by triggering their differentiation through an osteogenic lineage. We first showed that mrSCs isolated from a damaged muscle possess a higher osteogenic differentiation potential than cells from a control muscle. We then confirmed the hypoxic state of CTX-damaged skeletal muscle. As hypoxia in the microenvironment of mrSCs can affect their differentiation potential, we performed in vitro assays that showed that mrSCs respond to hypoxia by increasing their osteogenic differentiation potential and decreasing their adipogenic potential unlike in normoxic incubator conditions. Interestingly, hypoxia markedly activated Smad1/5/8 in mrSCs for up to 5 days while, in normoxic incubator conditions, Smad1/5/8 was inactive after 3 h. In response to hypoxia, mrSCs also upregulated the mRNA and protein expression of BMP9, a strong osteogenic inducer, whereas BMP2 and BMP7 mRNA expression were not affected. Taken together, our results suggest that the hypoxic state in damaged muscle can contribute to HO as a permissive environment by promoting the osteogenic differentiation of mrSCs.

## Methods

### Animals

Three- to- six-month-old male C57Bl/6 mice (Charles River, Saint-Constant, QC, Canada) were used. All procedures involving animals were approved by the Institutional Animal Care and Use Committee of Université de Sherbrooke (Protocol #141-11B/15B).

### Muscle injury

To induce muscle damage, 25 μL of 10 μM CTX (Latoxan, Valence, France) was injected intramuscularly into the tibialis anterior (TA) and gastrocnemius (Gas) muscles of anesthetized mice as described previously [19]. Contralateral TA and Gas muscles were injected with 25 μL of 0.9% saline solution. The mice were euthanatized, and the TA and Gas muscles were immediately harvested and processed for cell isolation and histological examinations or were flash frozen for molecular analyses. Flash-frozen muscles were mechanically crushed in liquid nitrogen using a mortar and pestle and were stored at − 80 °C until used to extract protein or RNA.

### Isolation and culture of primary muscle resident stromal cells

Muscle resident stromal cells (mrSCs) were isolated and cultured as previously described [19, 26, 27]. Briefly, damaged and contralateral healthy muscles were minced and were digested with 1 mg/mL of collagenase I (Sigma-Aldrich, Oakville, ON, Canada) for 45 min at 37 °C. The tissue slurries were washed with growth medium (GM) composed of Dulbecco’s modified Eagle’s medium—high glucose (DMEM) supplemented with 10% fetal bovine serum (FBS; Hyclone, Thermo Fisher Scientific, Ottawa, ON, Canada). The slurries were then poured successively through 70-μm and 50-μm cell strainers (BD Falcon, Mississauga, ON, Canada). Total cells were counted and were plated at a density of 7500 cells/cm^2^ in collagen-coated tissue culture dishes (BD). The cells were cultured in GM at 37 °C in normoxic incubator conditions (18.5% O_2_, 5% CO_2_ humidified standard incubator subjected to a pressure of 1 atm) [28, 29] or hypoxia 1% O_2_ atmosphere containing 5% CO_2_ (Thermo/Forma 3140 Series 2 Incubator). After 2 days, the medium was changed to remove non-adherent cells. The mrSCs were expanded as adherent cells, and the medium was replaced every 2–3 days.

### Colony-forming unit-fibroblast (CFU-F) assay

The number of stromal progenitors in healthy and damaged muscles was evaluated using a CFU-F. Isolated cells were diluted in GM, were plated in 60-mm collagen-coated dishes, and were cultured at 37 °C in a normoxic incubator condition containing 5% CO_2_ for 2 days in GM. The medium was changed to eliminate non-adherent cells. The adherent cells were then cultured for 12 days in normoxic incubator condition or hypoxia, and the medium was changed once. Colonies were fixed with 70% ethanol and were stained with 0.1% crystal violet (Sigma-Aldrich). Colonies with 50 or more cells were counted.

### ^3^H-thymidine incorporation assay

Freshly isolated mrSCs were plated in GM (250 cells/cm^2^), and after 2 days, 0.75 μCi of tritiated thymidine (methyl-^3^H-thymidine; Perkin Elmer, Waltham, MA, USA) was added to the medium and the cells were cultured for a further 24 or 72 h. The cells were then rinsed with PBS and were lysed with 0.1 N NaOH. ^3^H-thymidine incorporation was measured in the cell lysates using a Scintiverse BD Cocktail (Fisher Scientific) and a Tri-Carb LSC 2100TR scintillation counter (Perkin Elmer).

### Adipogenic and osteogenic differentiation

The osteogenic and adipogenic differentiation protocols were adapted from published protocols [30, 31] and are presented in Additional file 1: Table S1. Briefly, mrSCs were seeded at a density of 7500 cells/cm^2^ in 60-mm collagen-coated (Millipore, Billerica, MA, USA) tissue culture dishes in GM until they reached confluence. For osteogenic differentiation, the cells were cultured in osteogenic medium for 7 days with or without 1 nM rhBMP9 (R&D Systems, Minneapolis, MN, USA), an osteoinducer. To assess mineralization, the cell cultures were stained with Alizarin Red S to detect calcium deposits (Sigma; 40 mM, pH 4.1). For adipogenic differentiation, the cells were first cultured in an adipogenic induction medium for 2 days, followed by 6 days in an adipogenic growth medium. An Oil Red O solution (Sigma; 0.5% Oil red O in isopropyl alcohol) was used to detect triglycerides in the lipid droplets of mature adipocytes.

### Quantitative PCR and microarray analyses

Total RNA was extracted from flash-frozen crushed muscles or cultured cells using TRIzol® (Thermo Fisher Scientific). RNA (2 μg) was reverse-transcribed using Reverse Transcriptase Superscript II (Invitrogen). qPCR was performed using 50 ng of cDNA under the following conditions: a 5-min denaturation step at 95 °C followed by 40 cycles of 40 s at 95 °C, 40 s at 56 °C, and 40 s at 72 °C. qPCR assays were performed on a Rotor-Gene 6000 (Corbett Robotics, Eight Mile Plains, Australia) using iQSYBR Green Supermix (BioRad, Mississauga, ON, Canada). The results were analyzed using the 2^−ΔΔCT^ relative quantification method normalized to 18S. The primer sets are listed in Additional file 1: Table S2.

### Western blotting

Cells or tissues were lysed in RIPA buffer (0.5% NP-40, 0.1% SDS, 150 mM NaCl, 50 mM Tris-HCl, pH 7.5). Proteins were separated by SDS–PAGE, transferred to PVDF membranes (Millipore), and probed overnight at 4 °C with primary antibodies directed against HIF-1α (1:1000; Abcam Inc., Toronto, ON, Canada), BMP9 (1:2000, G-23; Santa-Cruz Biotechnology, Dallas, TX, USA), phospho-Smad1/5/8 (1:1000; Cell Signaling Technology Inc., Danvers, MA, USA), Smad1/5/8 (1:1000, H18; Santa-Cruz Biotechnology), GAPDH (1:1000, FL-335; Santa-Cruz Biotechnology), or β-actin (1:1000, AC-15; Sigma-Aldrich, Oakville, ON, Canada). Blots were washed in PBS-Tween and were incubated for 1 h at room temperature with a horseradish peroxidase-conjugated anti-rabbit or anti-mouse secondary antibody (1:5000; Biorad). The protein bands were revealed on Amersham Hyperfilm™ ECL sheets (GE Healthcare, Burnaby, BC, Canada) according to the manufacturer’s instructions. The blots were digitized, and the bands were quantified by densitometry using ImageJ software v1.45s [32].

### Quantification of alkaline phosphatase activity

Freshly isolated mrSCs were plated in collagen-coated 6-well plates. When the cells reached 90% confluence, the medium was changed to DMEM supplemented with 5% Horse Serum (HS), 1% antibiotics, and increasing concentrations of rhBMP2 or rhBMP9 (0.01, 0.05, 0.1, 0.5, 1, 5, 10, and 20 nM) and the cells were cultured for another 3 days. Cells cultured in plates without rhBMP2 or rhBMP9 were used as controls. Alkaline phosphatase (ALP) activity (SensoLyte-pNPP Alkaline Phosphatase Assay Kit; Anaspec, San Jose, CA, USA) was assayed according to the manufacturer’s instructions. The hydrolysis of a para-nitrophenylphosphate (pNPP) substrate was monitored by measuring the absorbance at 405 nm using a microplate reader (Molecular Devices, Sunnyvale, CA, USA). ALP activity was then calculated from a standard curve (0–100 ng/mL of pNPP). The concentrations were normalized to the total number of cells per well for each experimental condition. Briefly, before the cells were lysed, 5 μg/mL of Hoechst 33342 was added to the cell culture medium, and five representative images per well were acquired using a fluorescent microscope (Leica Microsystems Inc., Concord, ON, Canada) and a Retiga EX cooled color digital camera (Qimaging, Surrey, BC, Canada). The total number of nuclei was counted using an image analysis program developed using MatLab software R2007b (MathWorks, Natick, MA, USA). The ALP activity of each independent experiment was normalized to the control.

The dose–response relationship of ALP activity (ALP) was modeled with respect to the concentrations of BMPs ([BMP]) as a sigmoid dose–response function: $\mathrm{ALP}={\mathrm{ALP}}_{\mathrm{min}}+\frac{\left({\mathrm{ALP}}_{\mathrm{max}}-{\mathrm{ALP}}_{\mathrm{min}}\right)}{1+{\left(\frac{\left[\mathrm{BMP}\right]}{{\mathrm{EC}}_{50}}\right)}^{-\beta }}$. Dose–response profiles usually have three important areas: (1) threshold concentration, (2) exponential increase when the concentration exceeds the threshold, and (3) a plateau when the maximum effect is reached (ALP_max_). The effective concentration (EC_50_) is defined as the concentration at which the response is half that of the maximal possible response (ALP = ALP_max_/2), and *β* is the Hill coefficient, which is indicative of the responsiveness of cells toward the cytokine. The model parameters (ALP_max_, *β*, and EC_50_) were estimated using a genetic algorithm as previously described [33, 34]. The confidence intervals were determined using a bootstrap methodology. Briefly, residuals between experimental and model data were randomly redistributed to generate 500 new noisy datasets, and the genetic algorithm was used once again to estimate the corresponding value of each parameter. The confidence intervals were calculated for each parameter (*P*_avr_) from the resulting populations generated (${P}_{\mathrm{avr}}\pm 1.96\frac{\sigma }{\sqrt{n}}$).

### Detection of hypoxia

Hypoxia in damaged muscle was detected using a Hypoxyprobe™-1 Omni kit (NPI, Burlington, MA, USA) according to manufacturer instructions. Briefly, mice received one intraperitoneal injection (i.p.) of pimonidazole HCl solution (60 mg/kg of body weight). After 1 h, the damaged and contralateral healthy TA muscles were harvested and were frozen. Cryosections (7 μm) were fixed in 90% acetone (10 min at − 20 °C) and were blocked in PBS supplemented with 10% goat serum, 1% BSA, and 0.2% Triton® X-100. Pimonidazole (PIM) binding and myofibers were detected by overnight incubation at 4 °C with a rabbit anti-PIM primary antibody (1:200, PAb2627AP; Hypoxyprobe, Inc., MA, USA) and a rat monoclonal anti-laminin-2 primary antibody (1:500, 4H8–2; Sigma-Aldrich), respectively. The cryosections were rinsed in PBS-Tween and were incubated with Alexa Fluor® 594-conjugated goat anti-rabbit IgG secondary antibody (1:1000, Thermo Fisher Scientific) for PIM binding and Alexa Fluor® 647-conjugated goat anti-rat IgG secondary antibody (1:1000, Thermo Fisher Scientific) for myofibers. Primary antibodies were omitted as a control. Indirect immunofluorescence was assessed using an Axioskop 2 phase contrast/epifluorescence microscope (Carl Zeiss, Inc., Thornwood, NY, USA). Photomicrographic images were captured using a Retiga SRV cooled color digital camera (Qimaging) and were processed using Adobe Photoshop CS5 (Adobe Systems Inc., San Jose, CA, USA).

### Statistical analysis

Data are expressed as the means ± SEM of at least three independently performed experiments. Differences between normally distributed groups were compared using a Student’s paired *t* test for analyses of tissues from the same mouse and an unpaired *t* test for samples from cells or different mice. For the dose–response assays, an analysis of variance (ANOVA) was performed to make sure that the plateau had been reached. Since dose–response experiments frequently show heteroscedasticity, Box-Cox transformations were used when necessary before the ANOVA to obtain uniform variances [33]. Power calculations were performed with a 95% confidence interval and only differences with a *p* < 0.05 were considered significant (*p < 0.05; **p < 0.01; ***p < 0.001). Data accumulation and calculations were performed using Excel 2011 (Microsoft Corp., Redmond, WA, USA). GraphPad Prism 6.0c software™ (GraphPad Software Inc., La Jolla, CA, USA) was used for all the statistical analyses.

## Results

### Severely damaged skeletal muscle contains more mrSCs

**Fig. 1:**
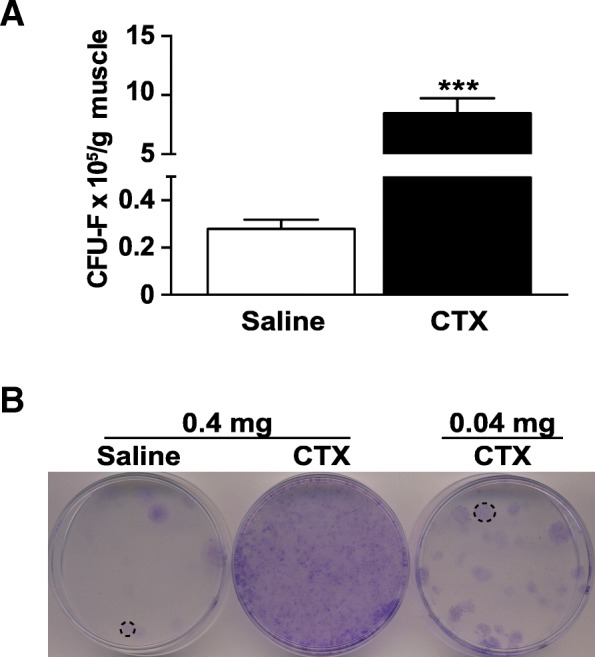
The mrSC population increases in damaged muscle. **a** Graph showing the number of progenitor mrSCs per gram of control and damaged muscle determined by a colony formation assay (CFU-F). There was a significant 30.3-fold increase in the number of mrSCs per gram of damaged muscle (CTX) compared to the control muscle (Saline) (mean ± SEM of four independent experiments; *n* = 4–6/experience; ***p < 0.001). **b** Photographs of representative CFU-F culture dishes of control (Saline) and damaged muscles (CTX). Each contains the equivalent of a 1:500 dilution (or 0.4 mg of muscle). For the damaged muscle, the number of CFU-F at this dilution was too high for them to be counted. A higher dilution was thus used (1:5,000 or 0.04 mg of muscle)

Following muscle damage, a regeneration process is triggered in which myogenic progenitors and mrSCs contribute to the repair of the myogenic and stromal portions of the muscle, respectively [35, 36]. As HO can form in damaged muscle and as mrSCs contribute to this process [19, 20, 37–39], the number of mrSCs was first determined in a damaged muscle and was compared to the number in a control muscle (saline injection). The tendency of mrSCs to adhere to plastic was used to determine their number in the control and damaged muscles using a colony-forming assay (Fig. 1a, b). The number of colonies (CFU-F) had increased more than 30-fold in the damaged muscle after 3.5 days, compared to control muscle (*p* < 0.001). This indicated that the population of mrSCs increases significantly within a few days of a severe muscle injury. These results are in accordance with our previous FACS data showing that there is an increase in the number of Sca1^+^ CD31^−^ Lin^−^ mrSCs per milligram of damaged muscle compared with control muscle [19].

### mrSCs from severely damaged skeletal muscle are prone to osteogenic differentiation

**Fig. 2:**
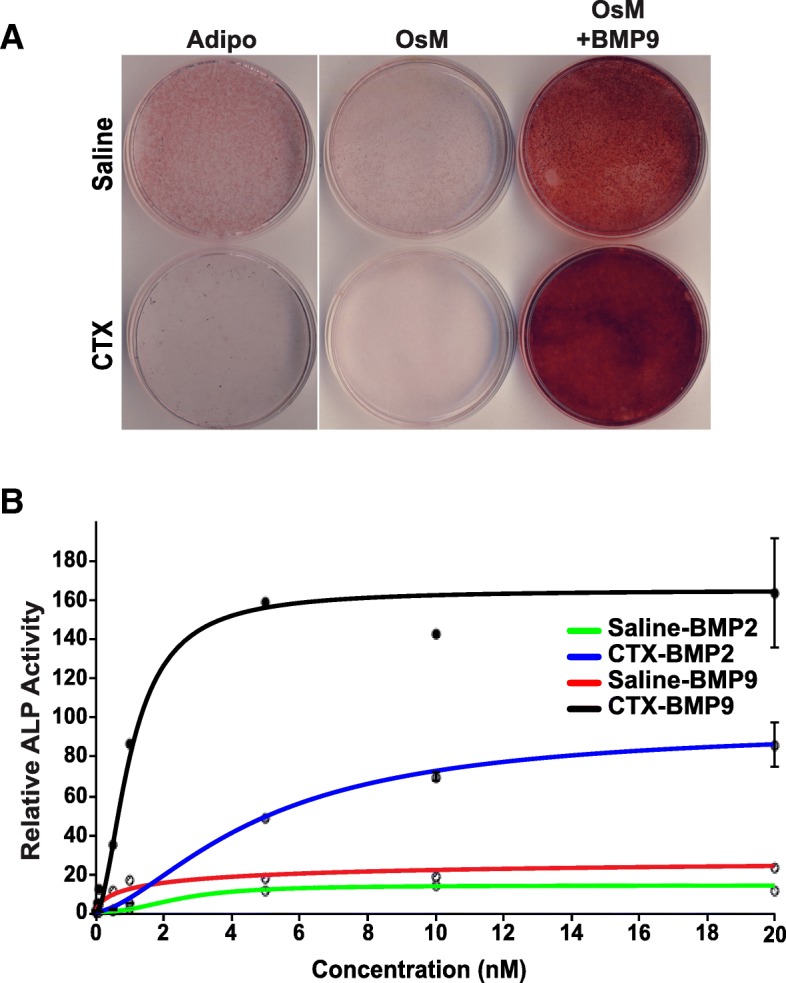
mrSCs from damaged muscle are more prone to osteogenic differentiation. **a** Representative micrographs of mrSCs isolated from control (Saline) and damaged (CTX) muscles cultured for 7 days in adipogenic (Adipo) or osteogenic (OsM) differentiation medium. mrSCs from the damaged muscle displayed a net decrease in adipogenic potential compared to those from the control muscle, as revealed by Oil Red O staining. In terms of osteogenic differentiation, no mineralization was observed in mrSCs from the control or the damaged muscle. However, the addition of 1 nM BMP9 to the osteogenic medium caused greater mineralization of mrSCs from the damaged muscle than of mrSCs from the control muscle, as revealed by Alizarin Red S staining. The results are representative of four independent experiments (*n* = 3–4/experiment). **b** Graph showing relative ALP activity (to untreated cells) of mrSCs isolated from the control (saline) and damaged (CTX) muscles treated for 3 days with increasing concentrations of BMP2 or BMP9 (mean ± SEM of a representative experiment, four independent experiments, *n* = 4–6/experiment). BMP9 induced a significant increase in maximum enzymatic activity compared to BMP2 for both cell preparations (saline and CTX). In addition, the minimum dose that induced a significant increase in ALP activity was lower with BMP9 than with BMP2. Lastly, mrSCs from the damaged muscle reacted more strongly to BMP9 and BMP2 than mrSCs from the control muscle. The dots represent experimental data while the lines represent the results of mathematical modeling of the ALP activity measurements

As mrSCs are multipotent progenitors, we determined whether the microenvironment of a damaged muscle can influence the ability of mrSCs to differentiate into adipocytes or osteoblasts, the cell types frequently encountered in regenerative deficits. mrSCs were isolated from control (saline) and CTX-damaged skeletal muscles 3.5 days post-treatment. They were cultured and differentiated in adipogenic medium (Adipo) or osteogenic medium (OsM) supplemented or not with 1 nM BMP9 (Fig. 2a). Oil Red O staining revealed that the adipogenic potential of mrSCs from the control muscle was enhanced while the adipogenic potential of mrSCs from the damaged muscle was reduced. On the other hand, the osteogenic potential of cells from the damaged muscle increased when the osteogenic medium was supplemented with BMP9 compared to mrSCs from the control muscle, as shown by the staining of mineral deposits by Alizarin Red S. Unlike the bone marrow MSCs (BM-MSC) (Additional file 1: Figure S1), the mineralization of the matrix only occurred when the mrSCs were cultured in an osteogenic differentiation medium supplemented with BMP9. These results suggest that osteoinducers play a critical role in the ability of mrSCs to differentiate into mature osteoblasts.

Our previous study showed that an intramuscular injection of BMP9 causes HO only in damaged muscle, suggesting that the microenvironment of damaged muscle modulates the response to BMP9, which induces mrSCs to form HO [19]. As mrSCs from damaged muscle display high osteogenic activity, we ascertained whether they have a better response to BMP9 by measuring the activity of alkaline phosphatase (ALP), an early differentiation marker involved in the mineralization process [33, 40] (Fig. 2b). We also ascertained whether the osteogenic response of mrSCs to BMP9 was comparable to the response to BMP2, an osteoinducer frequently used as a reference for osteogenic differentiation. ALP activity was measured in cells isolated from control (Saline) and damaged (CTX) muscles (3.5 days post-injury) after a 3-day incubation with increasing concentrations of BMP2 and BMP9 (0.01 to 20 nM). ALP activity was normalized to the total number of cells in each well. The results were modeled with respect to the concentration of BMP using the following equation: $\mathrm{ALP}={\mathrm{ALP}}_{\mathrm{min}}+\frac{\left({\mathrm{ALP}}_{\mathrm{max}}-{\mathrm{ALP}}_{\mathrm{min}}\right)}{1+{\left(\frac{\left[\mathrm{BMP}\right]}{{\mathrm{EC}}_{50}}\right)}^{-\beta }}$**.** The sigmoid profiles showed that ALP activity increased as a function of BMP concentration (basal activation), followed by an exponential increase when the BMP concentration exceeded the threshold and, finally, a plateau when the maximum effect was reached. An evolutionary-based optimization algorithm (genetic algorithm) was used to evaluate the parameters of the equation (ALPmax, EC_50_, and β) and the kinetic parameters of each curve (Saline/BMP2, Saline/BMP9, CTX/BMP2, CTX/BMP9) (Table 1). The *R*^2^ coefficients and *p* values of the different curves showed that the model faithfully represents the experimental results. The ALPmax parameter, which corresponds to the maximum response of the relative ALP activity (plateau), was evaluated for each condition. BMP9 caused a greater increase in the relative levels of ALPmax for both the control and damaged muscles (saline, 29.25 and CTX, 164.98) than BMP2 (saline: 14.47 and CTX: 95.22), suggesting that BMP9 is a stronger osteoinducer. In addition, the maximum response of ALP activity after a treatment with BMP9 was 5.6-fold (*p* < 0.001) higher for mrSCs from the damaged muscle than for mrSCs from the control muscle, while the response after a treatment with BMP2 was 6.6-fold (*p* < 0.001) higher for mrSCs from the damaged muscle than those from the control muscle. There was thus a strong and significant increase in the cellular response of mrSCs from the damaged muscle to the two BMPs. The half maximum effective concentration (EC_50_; ALP = ALPmax/2) was determined for each curve. The EC_50_ value for BMP9 (saline, 1.71 nM; CTX, 1.03 nM) was lower than that for BMP2 (saline, 2.46 nM; CTX, 4.81 nM), indicating that the cells responded more strongly to BMP9 than to BMP2, that is, at a lower concentration. Lastly, the minimum activation dose, i.e., the lowest concentration resulting in a significant increase in ALP activity, was determined for BMP2 and BMP9 and for cells isolated from control and damaged muscles. A dose of 0.05 nM BMP9 was sufficient to induce a significant increase in ALP activity in cells from the control muscle compared to a dose of 1 nM BMP2 (*p* < 0.001). On the other hand, a dose of 0.01 nM BMP9, the lowest concentration tested, caused a significant increase in ALP activity in cells from the damaged muscle compared to a dose of 1 nM of BMP2 (*p* < 0.05 and *p* < 0.001, respectively). These results indicate that cells in damaged muscle have a greater osteogenic potential in the presence of BMP than cells from healthy muscle. More importantly, a low dose of BMP9 produces greater osteogenic activity in mrSCs than BMP2, the reference osteoinducer.

**Table 1:** EC_50_ of BMPs and model parameters for the ALP activity of mrSCs isolated from control (Saline) and damaged (CTX) muscles

Parameters	Saline/BMP2	Saline/BMP9	CTX/BMP2	CTX/BMP9
*ALPmax*	14.47 ± 0.23	29.25 ± 0.51	95.22 ± 0.44	164.98 ± 0.48
*β*	2.40 ± 0.06	0.64 ± 0.04	1.59 ± 0.03	1.80 ± 0.03
EC_50_ (nM)	2.46 ± 0.07	1.71 ± 0.14	4.81 ± 0.02	1.03 ± 0.02
Minimum Effective Dose (nM)	1.0	0.05	1.0	0.01
*R* ^2^	0.9845	0.9591	0.9912	0.9884
*p* Value	< 0.001	< 0.001	< 0.001	< 0.001

### Severely damaged skeletal muscle is in a hypoxic state

**Fig. 3:**
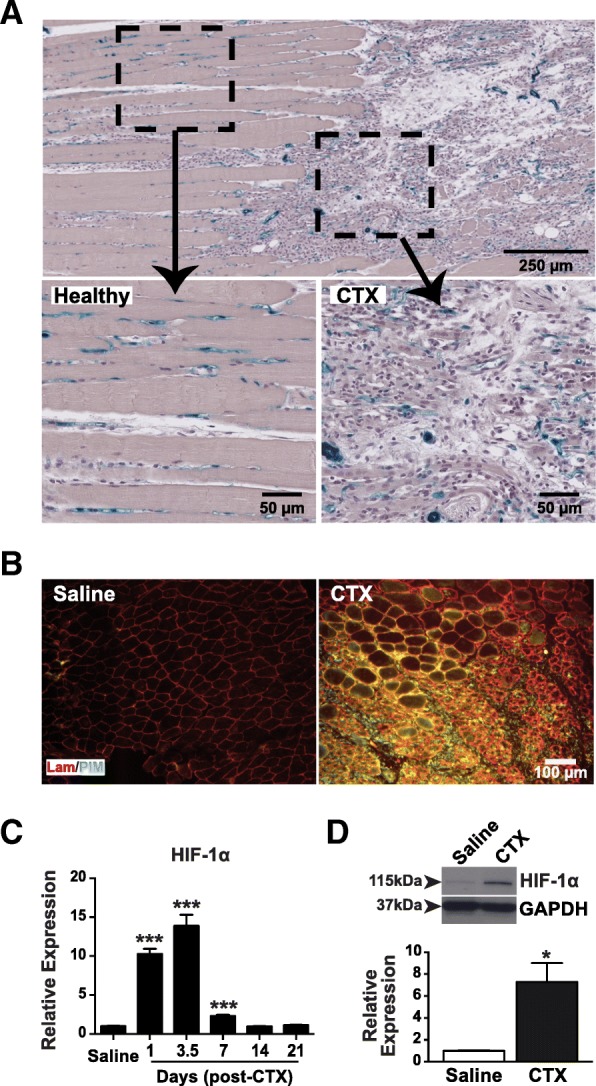
Damaged muscle is in a hypoxic state. **a** Representative micrograph of a longitudinal section of a damaged TA muscle (3.5 days post-CTX) from a TIE2lacZ mouse. A low magnification of the upper area of the muscle section shows an area where the architecture is typical of a healthy muscle and where muscle fibers and microvessels border on each other. The lower area has fewer muscle fibers as well as a significant number of nuclei resulting from an acute inflammatory cell infiltration characteristic of a damaged muscle. The lower micrographs show the organization of the two areas at a higher magnification. In the damaged area, the microvessels were fully disorganized. The results are representative of two independent experiments. **b** Frozen cross-sections of control (saline) and damaged (3.5 days post-CTX) TA muscles previously labeled with the hypoxic PIM probe showing hypoxia (green) and laminin (red) in the tissues. There is a hypoxic zone in the damaged muscle, unlike the control muscle. The micrographs are representative of three independent experiments. **c** Time course of relative mRNA expression of HIF-1α after a CTX-induced muscle injury. There is a significant increase in HIF-1α culminating 3.5 days post-CTX (13.9-fold) in the damaged muscle compared to the control muscle (saline) (mean ± SEM, *n* = 4; ***p < 0.001). **d** Western blot analysis of HIF-1α and an internal control (GAPDH) in the control (saline) and damaged (CTX) muscles. The expression of HIF-1α was 7.3-fold higher in the damaged muscle, which is in agreement with the increase in mRNA levels (mean ± SEM, *n* = 4; *p < 0.05)

CTX-induced injuries cause significant impairment of muscle structures. We observed longitudinal muscle sections from TIE2-lacZ mice by microscope 3.5 days after inducing CTX damage in muscles (Fig. 3a). The structure of the healthy part of the muscle had a parallel muscle fiber architecture. Microvessels stained blue with X-Gal were arranged along the muscle fibers. In the damaged part of the muscle, the muscle fibers were necrotic. Counterstaining with hematoxylin revealed a large number of nuclei, which is typical of significant leukocyte infiltration due to acute inflammation. The microvessels had also become disorganized along the damaged fibers. Taken together, these results suggest that significant inflammatory activity and microvessel disorganization causes higher O_2_ consumption and decreased O_2_ perfusion, resulting in a hypoxic microenvironment [25].

To investigate this possibility, control (saline) and damaged (3.5 days post-CTX) muscles were exposed to a PIM probe, which binds to hypoxic tissues [41]. A primary antibody that recognizes the probe and a fluorochrome-coupled secondary antibody were used to identify hypoxic sites. Laminin staining was also performed to identify the basement membranes of the muscle fibers (Fig. 3b). An analysis of muscle cross-sections showed that the probe was not immunolabeled in the control muscle but that substantial immunolabeling of the probe occurred between fibers and within degenerative fibers in the damaged muscle, indicating the presence of hypoxic sites. HIF-1α is a transcription factor that is regulated primarily by O_2_ concentrations [17, 42, 43] and is thus widely used as marker of hypoxia. To validate the results obtained with the PIM probe, the expression of HIF-1α mRNA was quantified in control and damaged muscles as a function of time post-damage (Fig. 3c). The damaged muscle exhibited a significant increase in HIF-1α mRNA levels as early as 24 h post-injury (10.3-fold; *p* < 0.001) and reached a maximum 3.5 days post-injury (13.9-fold; *p* < 0.001). HIF-1α mRNA levels were still significantly upregulated 7 days post-injury (2.3-fold; *p* < 0.001), but had returned to the basal level 14 and 21 days post-injury. Since HIF-1α is regulated by the proteasome [42, 43] and is thus independent of mRNA expression, HIF-1α protein levels were measured by Western blotting of protein extracts from the control and damaged muscles (3.5 days post-CTX) (Fig. 3d). There was a significant increase in HIF-1α protein levels in the damaged muscle compared with the control muscle (7.3-fold; *p* < 0.05). This increase was consistent with the mRNA and PIM probe results.

### Hypoxia increases the proliferation of mrSCs

**Fig. 4:**
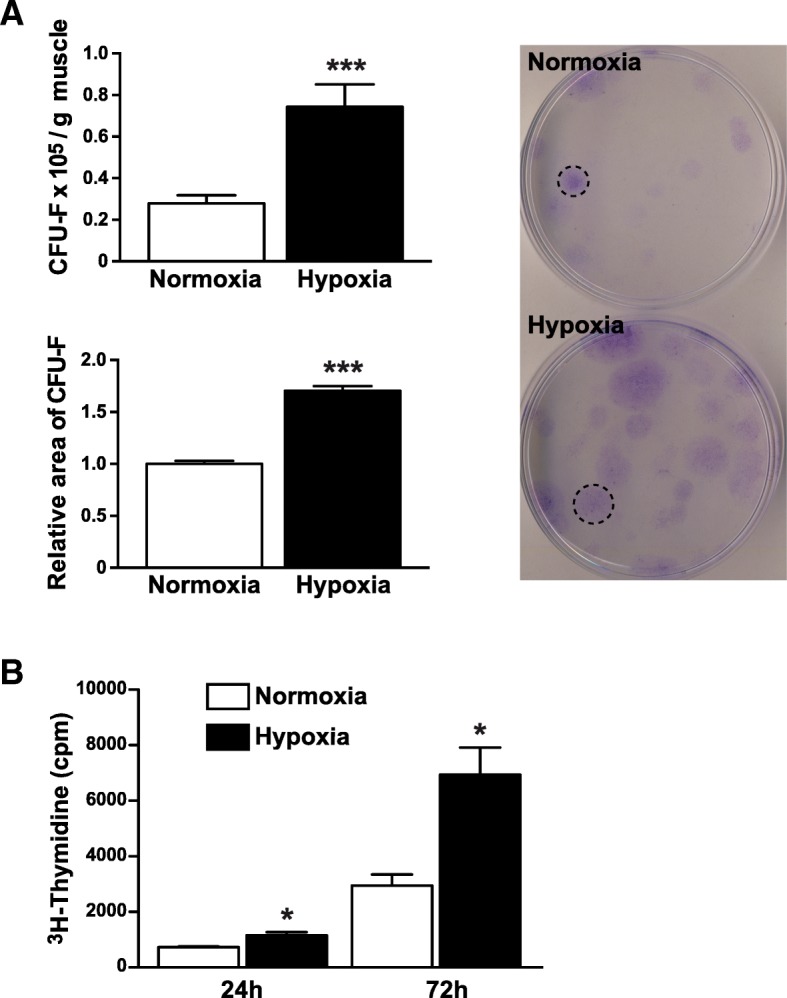
Hypoxia promotes the activation and proliferation of mrSCs. **a** CFU-F number and relative area of mrSCs isolated from the control muscle cultured in normoxic incubator condition (called “Normoxia”) or in hypoxia (1% O_2_). More cells were activated and proliferated, forming 2.7 times more colonies (> 50 cells), when they were cultured in hypoxia. In addition, colonies cultured in hypoxia were significantly bigger (1.7-fold) than cells cultured in normoxic incubator condition (mean ± SEM of four independent experiments; *n* = 4–6/experiment; ***p < 0.001). **b** Graph showing the incorporation of ^3^H-thymidine (cpm) by mrSCs cultured in hypoxia vs. mrSCs cultured in normoxic incubator condition. After 24 and 72 h, the cells cultured in hypoxia were significantly more proliferative than those cultured in normoxic incubator condition (1.6- and 2.4-fold, respectively) (mean ± SEM of two independent experiments; *n* = 3/experiment; *p < 0.05)

To determine whether hypoxia influences cell activation, mrSCs extracted from the healthy muscle were plated for a colony-forming assay. After 48 h in normoxic incubator condition (18.5% O_2_), debris and non-adherent cells were removed. Half the cells were then cultured in normoxic incubator condition and the other half were cultured in hypoxia for two weeks. Hypoxia was induced by reducing the concentration of O_2_ to 1%, a concentration sufficient to induce the hypoxia pathway (Additional file 1: Figure S2). Although the number of cells seeded was the same, a significant increase (2.7-fold; *p* < 0.001) in the number of CFU-F was observed with cells cultured in hypoxia compared to cells cultured in normoxic incubator condition (Fig. 4a). In addition, the significant increase in the size of the colonies of cells cultured in hypoxia (1.7-fold; *p* < 0.001) suggested that mrSCs have a higher proliferative capacity than when they are cultured in normoxic incubator condition. Since cell colony density can distort the interpretation of these results, a ^3^H-thymidine incorporation assay was performed (Fig. 4b). Cells cultured in hypoxia had incorporated 1.6- and 2.4-fold (*p* < 0.05) more ^3^H-thymidine after 24 and 72 h, respectively, than cells cultured in normoxic incubator condition, indicating that mrSCs cultured in hypoxia are significantly more proliferative than those cultured in normoxic incubator condition.

### Hypoxia affects the differentiation potential of mrSCs

**Fig. 5:**
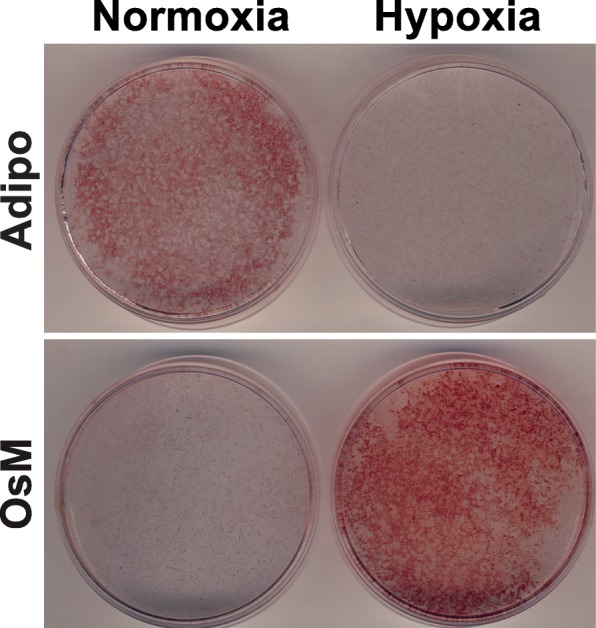
Hypoxia promotes the osteogenic differentiation of mrSCs. Representative micrograph of the in vitro adipogenic and osteogenic differentiation potentials of mrSCs that were isolated from control muscle and that were differentiated in normoxic incubator condition (called “Normoxia”) or hypoxia. In normoxic incubator condition, the mrSCs differentiated into adipocytes but not into osteoblasts, as indicated by Oil Red O and Alizarin Red S staining, respectively. On the other hand, hypoxia supported osteogenic differentiation at the expense of adipogenic differentiation. Osteogenic differentiation was possible even without the addition of an osteoinductive agent such as BMP9. The results are representative of four independent experiments (*n* = 3–4/experiment)

The differentiation potential of mrSCs from the damaged muscle was not the same as that of mrSCs from the control muscle (Fig. 2). We thus evaluated the influence of hypoxia on the multipotent differentiation capacity of these cells. mrSCs isolated from the healthy muscle were cultured in normoxic incubator condition until they reached 90% confluence. The medium was then replaced with adipogenic (Adipo) or osteogenic (OsM) differentiation medium, and the cells were cultured in normoxic incubator condition or in hypoxia throughout the differentiation process (Fig. 5). The adipogenic potential of mrSCs differentiated in hypoxia was greatly reduced as shown by the significant decrease in the number of Oil Red O-stained lipid droplets. On the other hand, hypoxia caused a marked increase in mineralization, as determined by Alizarin Red S staining, and consequently an increase in osteogenic potential. While the addition of an osteoinductive factor such as BMP9 was essential to cause the osteogenic differentiation and mineralization of mrSCs in normoxic incubator condition (for both control and damaged muscles), a hypoxic environment alone was sufficient to induce mineralization. Taken together, these results indicate that hypoxia promotes the osteogenic differentiation and mineralization of mrSCs without the addition of an osteoinductive factor.

### Hypoxia induces Smad1/5/8 phosphorylation and BMP9 expression in mrSCs

**Fig. 6:**
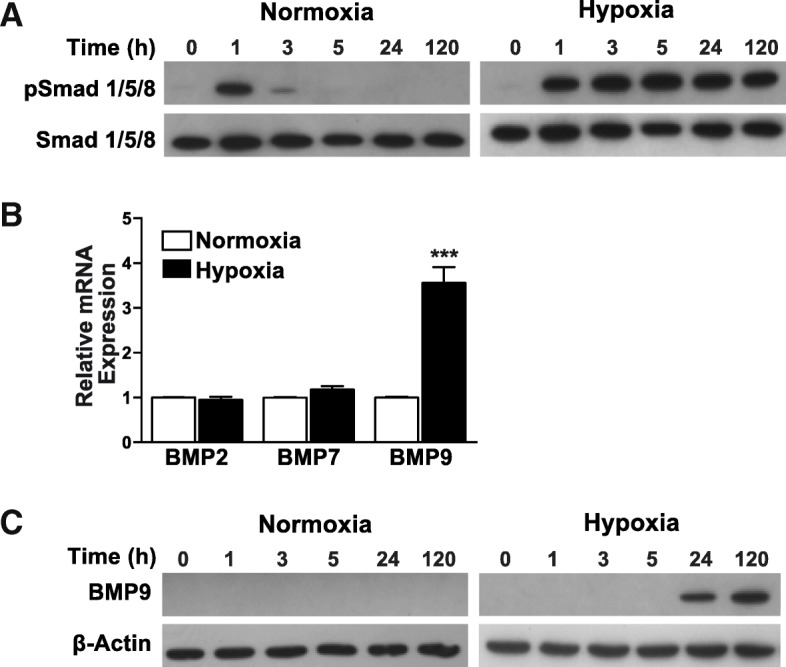
Hypoxia induces Smad1/5/8 phosphorylation and BMP9 expression in mrSCs. **a** Western blot analysis of phospho-Smad 1/5/8 and total Smad 1/5/8 in mrSCs cultured hypoxia or normoxic incubator conditions (called “Normoxia”). Hypoxia caused a marked and sustained activation of the Smad1/5/8 pathway even after 120 h. **b** Graph showing the expression of several osteogenic BMPs after a 24 h exposure to hypoxia. There was a significant increase (3.6-fold) in BMP9 expression by mrSCs cultured in hypoxia while the levels of BMP2 and BMP7 were not affected (mean ± SEM of four independent experiments; *n* = 4–6/experiment; ***p < 0.001). **c** Western blot analysis of BMP9 in mrSCs cultured in hypoxia or in normoxic incubator conditions. After 24 h of hypoxia, BMP9 was detected in mrSCs and was still expressed after 120 h

Effective osteogenic differentiation of mesenchymal progenitors occurs in the presence of BMPs via the phosphorylation of Smad1/5/8 [44, 45]. As a hypoxic environment in muscle has an impact on mrSCs in situ and their mineralization in vitro without the addition of an osteoinductive factor, it appears likely that hypoxia can promote the activation of the Smad pathway. The phosphorylation of Smad1/5/8 (pSmad1/5/8) is a key indicator of activation of the canonical pathway of BMPs [44, 45]. We used Western blot analyses to determine the levels of pSmad1/5/8 in mrSCs cultured in hypoxia and those cultured in normoxic incubator condition (Fig. 6a). The time course in normoxic incubator condition showed that pSmad1/5/8 levels spiked after the growth medium was replaced by a low serum medium after 1 h. pSmad1/5/8 levels decreased after 3 h, possibly due to the stress caused by the change of medium. Surprisingly, in the hypoxic culture condition, pSmad1/5/8 levels also increased after 1 h but remained elevated even after 120 h of culture. These results show that hypoxia can activate the BMP/Smad pathway and can also maintain a steady state of activation.

As BMP ligands can activate Smad signaling, we investigated the mRNA expression of several osteogenic BMPs (BMP2, BMP7, and BMP9) by qPCR in cells cultured in normoxic incubator condition or hypoxia for 24 h (Fig. 6b). Similar levels of BMP2 and BMP7 mRNA were observed in cells cultured in hypoxia and normoxic incubator condition. However, hypoxia significantly stimulated BMP9 mRNA expression (3.6-fold; *p* < 0.001). The expression of the BMP9 protein by mrSCs cultured in normoxic incubator condition and in hypoxia was also assessed over time (Fig. 6c). Western blot analyses revealed no detectable BMP9 protein in mrSCs cultured in normoxic incubator condition. However, BMP9 was detected in mrSCs cultured in hypoxia after 24 h, and high levels were detected after 5 days, indicating that hypoxia can induce mrSCs to produce endogenous BMP9, which is a strong osteogenic factor.

## Discussion

Much progress has been made in the past few years in better understanding the cellular and molecular causes of NHO and traumatic HO that involve progenitor cells, specific molecular signals directing the progenitor cell fate and a permissive environment [4, 6, 21, 46, 47]. For example, Kan et al. recently reported that Gli1-creERT-labeled cells contribute significantly to all stages of HO in adult triple transgenic mice (Nse-BMP4/Gli1-creERT-Zsgreen) and that these cells are also co-labeled by mesenchymal progenitor/stem cell markers, confirming that local tissue-resident mesenchymal progenitors/stem cells are involved in traumatic HO [48]. We and others have shown that mrSCs are involved in musculoskeletal trauma-induced HO and are able to transduce BMP signaling in vitro and in vivo to contribute to the development of HO [19, 20, 37–39]. While HO develops in damaged muscles with a permissive microenvironment, few studies have explored the impact of this permissive microenvironment on the phenotype of mrSCs and their ability to respond to inducing factors such as BMP9 [19, 38, 49, 50]. In the present study, the mrSC population not only expanded in the damaged muscle but also possessed a higher osteogenic potential in the presence of BMP9 than cells from the control muscle. This suggests that the microenvironment in damaged muscle activates normally quiescent mrSCs and alters their multipotency. We and others have previously reported that there is significant amplification of the mrSC population in mice and humans following injury [19, 38, 49–51]. For example, Davis et al. reported that there is a significant increase in the number of mrSC progenitors in the damaged muscles of patients suffering major combat-related trauma compared to the muscles of healthy patients and that these mrSCs also have a higher osteogenic potential [38]. This preferential differentiation may be caused by several factors that depend on muscle status (healthy vs. damaged) and thus on a specific microenvironment.

In a previous study, we showed that an inducing factor BMP9, unlike BMP2, induces HO in damaged muscle but not in healthy muscle [19]. We used quantitative measurements of ALP activity to show that mrSCs from damaged muscle are better to integrate signals from osteoinductive factors, in particular, BMP9, a “footprint” acquired from their original microenvironment. This may be due to the differences observed in the efficacy of BMP2 and BMP9 because they do not belong to the same BMP subfamily [19, 52] and do not use the same Ser/Thr kinase type I receptors. BMP2 binds predominantly to ALK3 receptors [53] while BMP9 has a high affinity for ALK1 receptors but also binds to ALK2 receptors [54, 55]. Although there are no known inhibitors of BMP9, BMP2 can be inhibited, for example, by Noggin and BMP3 [56, 57]. As such, a very small amount of BMP9 could have an osteogenic effect on mrSCs if they express the appropriate receptors. BMP9 can also act in synergy with specific cytokines and growth factors that are present in damaged muscle, promoting the initiation of the osteogenic differentiation. We have previously shown that the differentiation of murine preosteoblasts into osteoblasts increases when IGF-2 is added to a differentiation medium containing BMP9 [33]. Interestingly, IGF-2 mRNA levels increase significantly in damaged murine muscle [58].

We found using the PIM probe, i.e., that there is a loss of microvessel integrity after injury. The damaged muscle is also in a state of hypoxia as shown by significant increase in HIF-1α mRNA and protein levels in the damaged muscle compared with the control muscle. These results are in accordance with those of previous studies showing that there is a significant decrease in pO_2_ in damaged areas and increased HIF-1α levels (mRNA and protein) in the first hours or days following injury [23–25, 59]. For example, Scherrer et al. used PIM immunofluorescence staining to show that skeletal muscle enters a state of hypoxia 3–24 h after a crush injury. They also used an immunohistochemical approach to detect HIF-1α, the principal mediator of the hypoxic response, in damaged muscle, particularly in inflammatory cells that invade the muscle [25]. In fact, during the inflammatory phase, neutrophils and macrophages use a large amount of oxygen to produce H_2_O_2_ and reactive oxygen species (ROS), a process called respiratory burst [60–62]. In summary, the alteration in the organization of microvessels coupled with acute inflammation contributes to the setting up of hypoxic conditions in damaged muscle. This condition is temporary as the resolution of inflammation and the reorganization of the microvascular network occurs approximately 8–12 days after the injury [63, 64].

Hypoxia plays an important role in regulating endochondral bone formation [13, 65, 66]. Wang et al. used a mouse model that overexpresses HIF-1α in osteoblasts to show that HIF-1α mice develop high-density long bones while HIF-1α-deficient mice have thinner long bones [66]. A number of studies have shown that hypoxia is involved in the development of HO [9–11, 67, 68]. Agarwal et al. used three different models of trauma-induced HO to show that HIF-1α is present during immature HO [68]. We showed that there is an increase in the activation and proliferation of mrSCs and that osteogenic differentiation is promoted at the expense of adipogenic differentiation when cells from healthy muscle are exposed to a hypoxic environment. Hypoxic state in damaged muscle thus contributes to an alteration of the mrSC phenotype. Oxygen is an important component of the microenvironment of MSCs and previous studies have shown that its concentration can affect their phenotype [31, 69, 70]. Wagegg et al. reported that primary human MSCs that were isolated from bone marrow and that were cultured 2 weeks in a hypoxic chamber were unable to differentiate into an adipogenic lineage while osteogenic differentiation was not affected [69]. Hypoxia also inhibits the expression of PPARγ in preadipocytes, preventing their differentiation into adipocytes [71]. This might explain the inhibition of adipogenic differentiation we observed in mrSCs from damaged muscle and in mrSCs cultured in hypoxic conditions. Hung et al. also reported that hypoxia favors the proliferation of human BM-MSCs and increases their osteogenic potential but reduces their adipogenic potential [31]. However, these results were contradicted by a recent study by Agarwal et al., who reported that the proliferation of human BM-MSCs decreases in hypoxic conditions. Despite this contradiction, they noticed an increased osteogenic potential when they maintained cells in a hypoxic environment. [72].

During the osteogenic differentiation of mrSCs in vitro, a hypoxic environment was sufficient to induce mineralization, removing the need for exogenous osteoinducer. We observed a persistent phosphorylation of Smad1/5/8 within 5 days when mrSC were cultured in hypoxia. The Smad1/5/8 pathway plays an important role in osteogenic differentiation [44, 45]. Wang et al. showed that FOP SHED cells (stem cells from human exfoliated deciduous teeth) respond to hypoxia with elevated levels of Smad1/5/8 phosphorylation [11, 46]. They also reported that hypoxia increases the intensity and duration (24 h) of canonical BMP signaling in the absence of an exogenous BMP ligand and that Noggin has no effect on Smad1/5/8 phosphorylation in FOP SHED cells. Furthermore, the inhibition of HIF-1α by the specific PX478 inhibitor restores canonical BMP signaling to normoxic levels and reduces HO in a mouse model of FOP [11]. Our results also showed that the osteoinductive Noggin-resistant factor BMP9, but not BMP-2 or BMP-7, is synthesized by mrSCs in hypoxic conditions. BMP9 is endogenously produced by osteoblasts and stromal cells in human HO [22]. In addition, Hirata et al. used a microarray approach to analyze 522 mRNA profiles expressed during the regeneration of damaged TA muscle and reported that the expression of 40 genes increased more than fivefold 48 h post-injury and 64-fold 96 h post-injury. These genes included various proinflammatory receptors as well as BMP9 [58]. In the present study, we showed that BMP9 is endogenously expressed by mrSCs after 24 h in hypoxia. This endogenously expressed BMP9 might be also involved in their osteogenic differentiation observed in hypoxic culture condition. Hu et al. used immortalized mouse embryonic fibroblasts co-transduced with BMP9 and an HIF-1α adenovirus injected subcutaneously into the flanks of nude mice to show that BMP9-induced ectopic bone formation is enhanced by HIF-1α expression and that it is reduced when HIF-1α is silenced [73]. These observations, combined with our results, suggest that the hypoxia pathway and its chief mediator, HIF-1α, play important roles in the development of HO.

In the present study, we found that hypoxia, a permissive environment for the development of HO, can promote mrSC differentiation into osteoblasts, induce Smad1/5/8 phosphorylation and BMP9 expression in mrSC. Furthermore, mrSCs from damaged muscle amplified their response to exogenous BMP9 compared to BMP-2. Interestingly Salga et al. have recently shown that NHO formation at 21 days in mice hamstring muscle requires not only a spinal cord injury combined with botulinum toxin A treatment but also a cardiotoxin-damaged muscle [74]. This observation agreed well with the study of Genet et al. that found NHO in animal with spinal cord transection and intramuscular cardiotoxin injection [75]. It will be therefore quite interesting in a future work to determine the effect of a spinal cord injury in the HO animal model, we have used in the present study (damaged muscle induced by cardiotoxin injection), on the mrSCs behaviors and endogenous-inducing factors availability and efficacy.

## Conclusion

In summary, we show how the specific microenvironment in damaged muscles influences the proliferation and differentiation of mrSCs, which are involved in the trauma-induced HO. We also show that oxygen, which is involved in numerous physiological and pathological processes, influences the behavior of mrSCs by promoting their activation and proliferation and also by increasing their capacity to form bone. This effect may be mediated by the activation of the Smad pathway and by the expression of BMP9 by mrSCs, but the underlying mechanisms still need to be studied. A better understanding of the permissive environment effect on the progenitor cell fate as well as inducing factor efficacy in the development of HO (NHO and traumatic HO) is essential to create future therapies.

## Additional file

Additional file 1: **Figure S1.** Comparison of the osteogenic potential of mrSCs and BM-MSCs. **Figure S2.** In vitro induction of HIF-1α activity. **Table S1.** Compositions of the osteogenic and adipogenic differentiation media. **Table S2.** Primer sets used for the qPCR. (DOCX 93 kb)

Additional file 2: Mathematical modeling of the dose response relationship of ALP activity with respect to the BMP concentrations. (XLSX 726 kb)

## Data Availability

The datasets used and/or analyzed during the current study are available from the corresponding author on reasonable request. The mathematical modeling of the dose–response relationship of ALP activity (ALP) with respect to the concentrations of BMPs is provided as Additional file 2.

## Abbreviations

ALP: Alkaline phosphatase
BM-MSC: Bone marrow mesenchymal stem cells
BMP: Bone morphogenetic protein
CFU-F: Colony-forming unit-fibroblast assay
CTX: Cardiotoxin
FOP: Fibrodysplasia ossificans progressiva
Gas: Gastrocnemius muscle
GM: Growth medium
HIF: Hypoxia-inducible factors
HO: Heterotopic ossification
HRE: Hypoxia-responsive element
mrSCs: Muscle resident stromal cells
NHO: Neurogenic heterotopic ossification
PIM: Pimonidazole
pNPP: para-nitrophenylphosphate
TA: Tibialis anterior muscle
