# Supplementary material for: Muscle injury-induced hypoxia alters the proliferation and differentiation potentials of muscle resident stromal cells
Source: Skelet Muscle. 2019 Jun 19;9:18. doi: 10.1186/s13395-019-0202-5 (PMC6582603; doi:10.1186/s13395-019-0202-5)
Supplement: Supplementary file 1 — Figure S1. Comparison of the osteogenic potential of mrSCs and BM-MSCs. Figure S2. In vitro induction of HIF-1α activity. Table S1. Compositions of the osteogenic and adipogenic differentiation media. Table S2. Primer sets used for the qPCR. (DOCX 93 kb) [file 13395_2019_202_MOESM1_ESM.docx]

**Supplemental Data**

**Materials & Methods**

***Measurement of HIF-1α*** ***transcriptional activity using a luciferase assay***

The activity of the hypoxia pathway was measured using the HRE-luciferase reporter plasmid (26731; Addgene, 26731; Cambridge, MA, USA). Freshly isolated mrSCs were transfected with 200 µg of HRE-luciferase plasmid 3 days after being seeded in a 24-well plate in serum-free DMEM containing 1 µL of Fugene 6™ (Promega, Madison, WI, USA) and were cultured for 24 h. The medium was replaced with DMEM supplemented with 5% (v/v) HS and 1% (v/v) P/S and the mrSCs were cultured for a further 24 h. They were then cultured for 6 h or 24 h in normoxic incubator conditions or in hypoxia. The transcriptional activity of the hypoxia pathway was measured using a Luciferase Assay System kit (Promega) and a Titertek-Berthold luminometer (Pforzheim, Germany). The data were normalized to the protein content measured using the Bradford method.


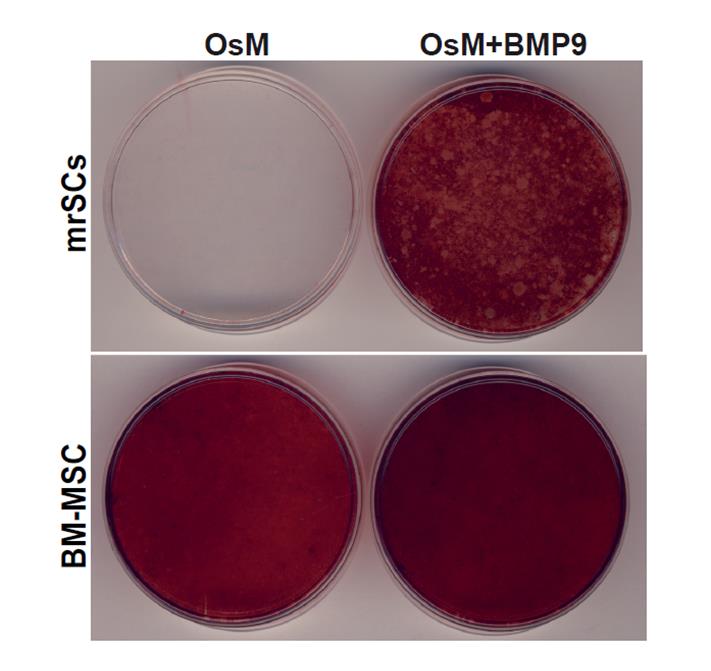


**Figure S1. Comparison of the osteogenic potential of mrSCs and BM-MSCs**

Representative micrographs of the *in vitro* osteogenic differentiation of mrSCs and BM-MSCs with or without BMP9. Without BMP9, only the BM-MSCs differentiated into osteoblasts, as revealed by Alizarin Red S staining. When 1 nM of BMP9 was added to the osteogenic medium, the mrSCs also differentiated into osteoblasts.


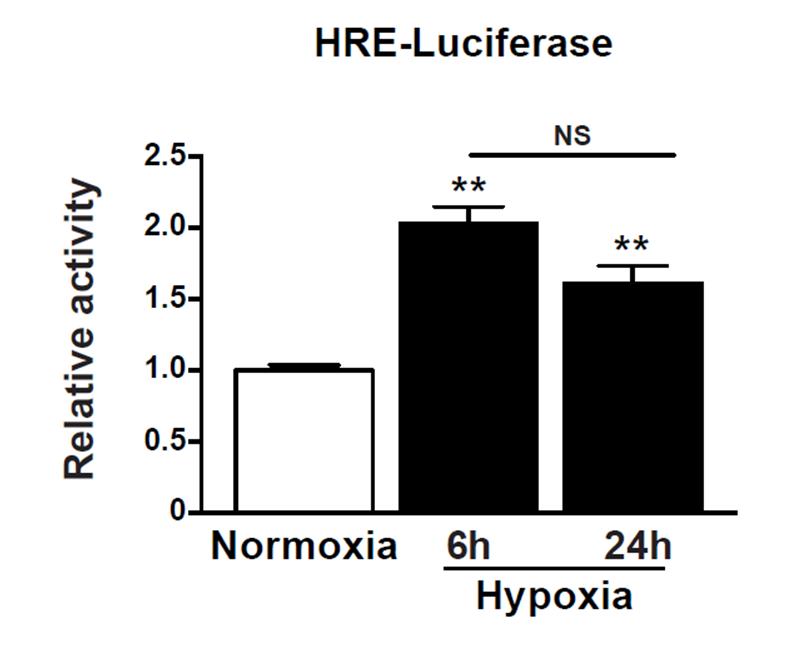


**Figure S2. *In vitro* induction of HIF-1α activity**

The transcriptional activity of HIF-1α was measured as a function of time. The reporter plasmid HRE-luciferase was transfected into mrSCs cultured in normoxic incubator conditionsor hypoxia (1% O_2_). There was a significant increase in luciferase activity in mrSCs cultured in hypoxia after 6 and 24 h, indicating that 1% O_2_ is sufficient to activate the HIF-1α pathway in mrSCs (mean ± SEM of two independent experiments, n=3/experiment; **p<0.01).

**Table S1. Compositions of the osteogenic and adipogenic differentiation media**

| **Type** | **Basal Medium** | **Supplements** | **Staining** |
| --- | --- | --- | --- |
| **Osteogenic** | DMEM, 5% (v/v) HS | 20 µg/ml ascorbic acid | Alizarin Red S |
|  | 1% (v/v) antibiotics | 100 nM dexamethasone |  |
|  |  | 10 mM β-glycerophosphate |  |
| **Adipogenic**  **(Induction)** | DMEM, 10% (v/v) FBS | 1 µM dexamethasone |  |
|  | 1% (v/v) antibiotics | 5 µg/mL insulin |  |
|  |  | 0.5 mM isobutyl methylxanthine (IBMX) |  |
| **Adipogenic** | DMEM, 10% (v/v) FBS | 5 µg/ml insulin | Oil red O |
|  | 1% (v/v) antibiotics |  |  |

**Table S2. Primer sets used for the qPCR**

| **Gene** | **Forward Primer** | **Reverse Primer** | **NM NCBI** |
| --- | --- | --- | --- |
| BMP2 | TGGAAGGTGGCCCATTTAGAG | TGACGCTTTTCTCGTTTGTG | NM_001200 |
| BMP7 | GAAAACAGCAGCAGTGACCA | GGTGGCGTTCATGTAGGAGT | NM_001719 |
| BMP9 | GGCTGAGCTCCGACTCTATG | AACCTGGAGGGACACTGATG | NM_016204 |
| HIF-1α | TCAAGTCAGCAACGTGGAAG | TATCGAGGCTGTGTCGACTG | NM_010431 |
| 18S | AGGAATTGACGGAAGGGCAC | CGACATCTAAGGGCATCACA | NR_003286 |
